# Complete Genome Sequences of Rhizobium leguminosarum bv. *phaseoli* BT01, *Rhizobium* sp. Strain BT03, and *Rhizobium* sp. Strain BT04, Isolated from Black Turtle Bean Nodules in Ontario, Canada

**DOI:** 10.1128/mra.00445-23

**Published:** 2023-06-20

**Authors:** Sabhjeet Kaur, Catherine E. Christie, George C. diCenzo

**Affiliations:** a Department of Biology, Queen’s University, Kingston, Ontario, Canada; b KASSI (Kingston Area Seed System Initiative), Kingston, Ontario, Canada; The University of Arizona

## Abstract

We report the complete genome sequences of three *Rhizobium* strains isolated from nodules of heritage black turtle bean (Phaseolus vulgaris) plants grown in a community garden in Ontario, Canada. The genomes are between 6.91 Mb and 7.98 Mb long and consist of five to seven DNA replicons.

## ANNOUNCEMENT

The common bean (Phaseolus vulgaris) was introduced to southern Ontario by Indigenous communities prior to CE 1250 (1, 2). Southern Ontarian soils may therefore represent a source of novel P. vulgaris rhizobial symbionts able to support bean production in Canada. We isolated rhizobia from three nodules collected from two black turtle bean plants grown at a community garden located in Kingston (Ontario, Canada) in October 2022. The black turtle bean is native to Mexico, and its cultivation began spreading throughout North America in the mid-1800s (3).

Bacterial isolation from nodules was performed as described previously (4) Isolates were streak purified three times on tryptone-yeast extract (TY) medium (4), and single colonies were then used to prepare 7% dimethyl sulfoxide (DMSO) stocks for storage at −80°C. For DNA isolation, single colonies were inoculated into TY broth and grown overnight at 28°C, following which DNA was extracted using Monarch genomic DNA purification kits (New England Biolabs) according to the manufacturer’s instructions. Oxford Nanopore Technologies (ONT) sequencing was performed using a rapid barcoding kit (SQK-RBK004; ONT) and a R9.4.1 flow cell on a MinION device. Base calling and demultiplexing were performed using Guppy version 6.4.6+ae70e8f with the r941_min_sup_g507 model (ONT). Illumina sequencing was performed on a NextSeq 2000 instrument at SeqCenter (Pittsburgh, PA, USA) using the Illumina DNA prep kit and IDT 10-bp unique dual indexes (UDI), producing 2 × 151-bp reads. The Illumina reads were filtered using BBDuk version 38.96 (5) and trimmed using Trimmomatic version 0.39 (6), with the following parameters: LEADING:3 TRAILING:3 SLIDINGWINDOW:4:15 MINLEN:36. The sequencing statistics are provided in Table 1.

**TABLE 1:** Accession numbers and sequencing, assembly, and annotation statistics

Feature	Data for strain:		
	Rhizobium leguminosarum bv. *phaseoli* BT01	*Rhizobium* sp. BT03	*Rhizobium* sp. BT04
BioProject accession no.	PRJNA967359	PRJNA967359	PRJNA967359
BioSample accession no.	SAMN34588606	SAMN34588607	SAMN34588608
GenBank Assembly accession no.	GCF_030053175.1	GCF_030053155.1	GCF_030053135.1
GenBank accession no.	CP125635, CP125636, CP125637, CP125638, CP125639	CP125640, CP125641, CP125642, CP125643, CP125644, CP125645, CP125646	CP125647, CP125648, CP125649, CP125650, CP125651, CP125652, CP125653
SRA accession no.			
ONT reads	SRR24561042	SRR24561041	SRR24561040
Illumina reads	SRR24561045	SRR24561044	SRR24561043
ONT data			
Total read length (nt)^*a*^	2,378,429,724	1,247,169,810	1,394,437,248
No. of reads	708,175	240,137	345,171
*N*_50_ read length (nt)	6,092	10,303	7,533
Illumina data			
Total read length (nt)	520,709,902	400,009,796	704,473,851
No. of paired reads	1,966,528	1,534,831	2,701,212
Read length (nt)	2 × 151	2 × 151	2 × 151
Genome size (bp)	7,975,513	6,907,391	6,989,060
No. of protein coding genes	7,360	6,357	6,403
G+C content (%)	60.50	61.63	61.02
No. of replicons	5	7	7
Replicon sizes (bp)	4,892,895, 1,334,842, 652,849, 631,455, 463,472	4,499,098, 654,550, 559,700, 446,819, 326,235, 293,100, 127,889	4,676,374, 659,089, 540,617, 422,655, 274,203, 216,650, 199,472

a nt, nucleotides.

Draft genome sequences for Rhizobium leguminosarum bv. *phaseoli* BT01 and *Rhizobium* sp. strain BT04 were generated from the ONT reads using Flye version 2.9-b1779 (7). As Flye did not produce a fully circularized genome for *Rhizobium* sp. BT03, a *Rhizobium* sp. BT03 draft genome was instead generated using Unicycler version 0.5.0 (8) with SPAdes version 3.15.4 (9) and the ONT and Illumina reads. The assemblies were then polished using the ONT reads and Medaka version 1.7.2 (ONT). The assemblies were further polished using the Illumina reads, first using Polypolish version 0.5.0 (10) and then using POLCA version 4.0.9 (11), with read mapping performed using bwa version 0.7.17-r1198-dirty (12). The fixstart option of Circlator version 1.5.5 (13) was used to reorient replicons to start at the *dnaA* gene, if found, or otherwise at a gene nearest to the middle of the replicon. Finally, the genomes were annotated using PGAP version 2022-10-03.build6384 (14). Eight threads (when multithreading was possible) and default parameters were used for all software unless otherwise specified. Assembly and annotation statistics are provided in Table 1.

**FIG 1:**
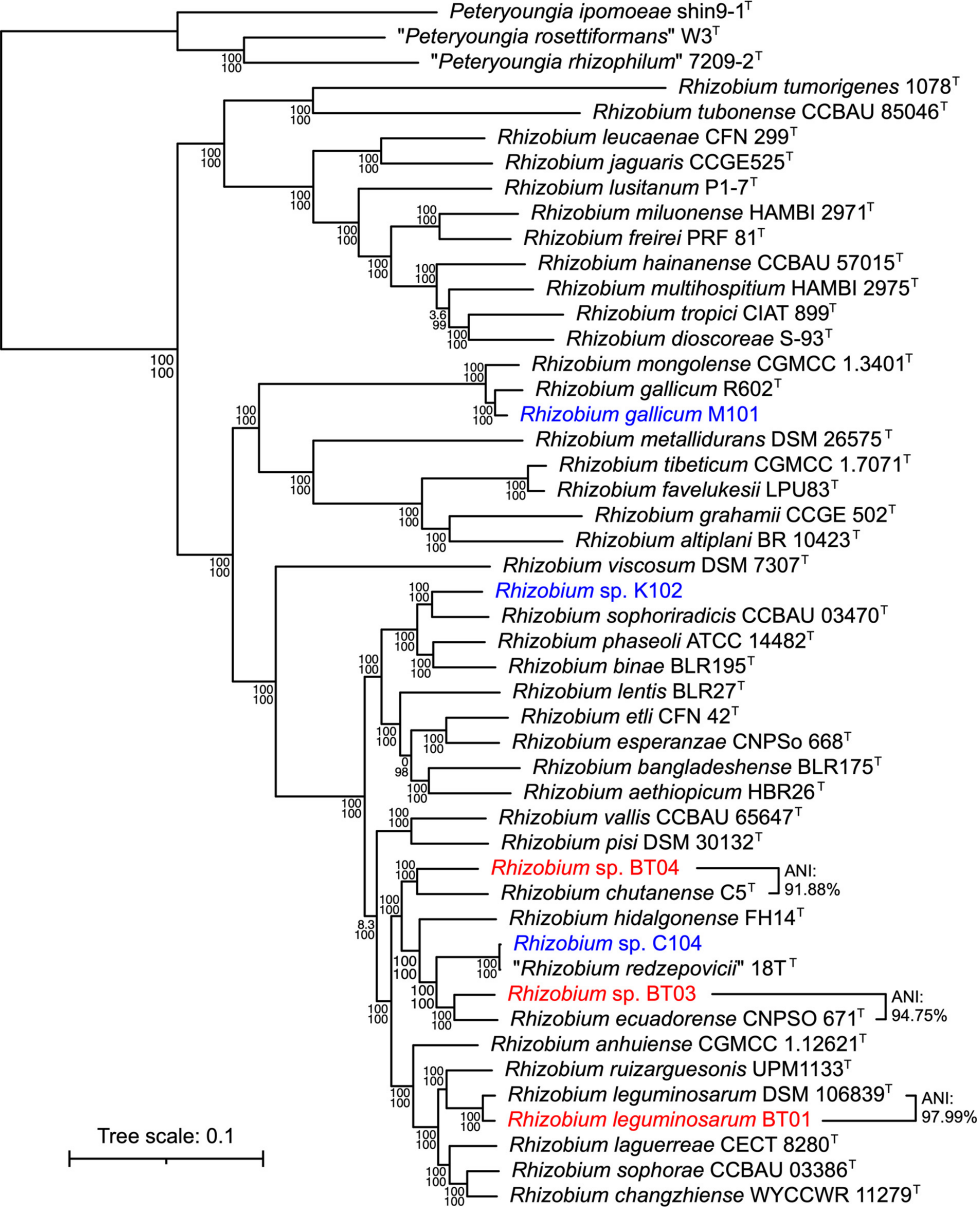
Maximum likelihood phylogeny of the genus *Rhizobium*. A maximum likelihood phylogeny of 45 *Rhizobium* strains was prepared from a concatenated alignment of 646 core genes. Three *Peteryoungia* strains were included as an outgroup. Strains sequenced as part of this study are shown in red, while three bean symbionts recently isolated in Manitoba, Canada (17), are shown in blue. To construct the phylogeny, Roary version 1.7.8 (18) was used to identify core genes (80% identity threshold) and to create a concatenated alignment using MAFFT version 7.471 (19). The concatenated alignment of 646 genes was trimmed using trimAl version 1.4.rev22 (20) and used to construct a maximum likelihood phylogeny using IQ-TREE version 2.2.0 and the GTR+F+I+I+R6 model (21). Two values are given at each node. The top number represents the Shimodaira-Hasegawa-like approximate likelihood ratio test (SH-aLRT) support value, calculated from 1,000 replicates (22). The bottom number represents the ultrafast jackknife support value calculated from 1,000 replicates and a subsampling proportion of 40%. The scale represents the mean number of nucleotide substitutions per site. Average nucleotide identity values are given on the right side of the figure for comparisons between the newly isolated strains and the most closely related species type strains.

To taxonomically classify the three novel strains, a core genome maximum likelihood phylogeny was constructed as described previously (Fig. 1) (15), and average nucleotide identities (ANI) were calculated against 39 *Rhizobium* species type strains using FastANI version 1.33 (16). These results indicated that strain BT01 belongs to the species R. leguminosarum, while BLAST searches of the NodA, NodB, and NodC proteins against the National Center for Biotechnology Information nonredundant database assigned strain BT01 to the biovar *phaseoli*. *Rhizobium* sp. BT03 could not be unambiguously assigned to a species but potentially belongs to Rhizobium ecuadorense. *Rhizobium* sp. BT04 appears to represent a novel species, as all ANI values were <92%, but it is most closely related to Rhizobium chutanense. In addition, our three bean symbionts differed from not only each other but also those recently isolated in Manitoba, Canada (17), suggesting that there is a large reservoir of untapped bean symbiont biodiversity in Canada.

### Data availability.

The annotated genome assemblies and raw sequencing reads were deposited at the GenBank assembly and Sequence Read Archive databases, respectively, and are accessible via the accession numbers listed in Table 1. Scripts to repeat the genome assembly and annotation, as well as the average nucleotide identity and phylogenetic analyses, are available through GitHub at https://github.com/diCenzo-Lab/009_2023_Black_Turtle_Bean_rhizobia.
